# Kilocalorie labelling in the out-of-home sector: an observational study of business practices and consumer behaviour prior to implementation of the mandatory calorie labelling policy in England, 2022

**DOI:** 10.1186/s12889-023-16033-8

**Published:** 2023-06-06

**Authors:** Megan Polden, Andrew Jones, Jean Adams, Tom Bishop, Thomas Burgoine, Michael Essman, Stephen J. Sharp, Richard Smith, Martin White, Eric Robinson

**Affiliations:** 1 Department of Primary Care & Mental Health, University of Liverpool, Liverpool, UK; 2 Liverpool John Moores University, Liverpool, UK; 3 MRC Epidemiology Unit, University of Cambridge, Cambridge, UK; 4 College of Medicine and Health, University of Exeter, Exeter, UK; 5 Department of Psychology, University of Liverpool, Eleanor Rathbone Building, Bedford Street South, Liverpool, L69 7ZA UK

**Keywords:** Energy labelling, Calorie labels, Obesity policy, Out-of-home food sector

## Abstract

**Background:**

Regulations mandating kilocalorie (kcal) labelling for large businesses in the out-of-home food sector (OHFS) came into force on 6th April 2022 as a policy to reduce obesity in England. To provide indicators of potential reach and impact, kcal labelling practices were studied in the OHFS, and customer purchasing and consumption behaviours prior to implementation of the mandatory kcal labelling policy in England.

**Methods:**

From August-December 2021, large OHFS businesses subject to the kcal labelling regulations were visited prior to regulations coming into force on 6th April 2022. 3308 customers were recruited from 330 outlets and collected survey information on the number of kcal purchased and consumed by customers, customers’ knowledge of the kcal content of their purchases, and customers noticing and use of kcal labelling. In a subset of 117 outlets, data was collected on nine recommended kcal labelling practices.

**Results:**

The average number of kcals purchased (1013 kcal, SD = 632 kcal) was high with 69% of purchases exceeding the recommendation of a maximum of 600 kcal per meal. Participants underestimated the energy content of their purchased meals by on average 253 kcal (SD = 644 kcals). In outlets providing kcal labelling in which customer survey data was collected, a minority of customers reported noticing (21%) or using (20%) kcal labelling. Out of the 117 outlets assessed for kcal labelling practices, 24 (21%) provided any in-store kcal labelling. None of the outlets met all nine aspects of recommended labelling practices.

**Conclusions:**

Prior to implementation of 2022 kcal labelling policy, the majority of sampled OHFS large business outlets in England did not provide kcal labelling. Few customers noticed or used the labels and on average customers purchased and consumed substantially more energy than recommended in public health guidelines. The findings suggest that reliance on voluntary action for kcal labelling implementation failed to produce widespread, consistent, and adequate kcal labelling practices.

**Supplementary Information:**

The online version contains supplementary material available at 10.1186/s12889-023-16033-8.

## Introduction

The out of out-of-home food sector (OHFS) refers to outlets where food or drink is prepared for immediate consumption, on or off the premises and includes outlets such as sit-down restaurants, cafes, and takeaways [1]. Consuming food from OHFS is becoming increasingly common in the UK with 25–39% of adults consuming such food at least once a week, based on data collected in 2008-12 and 2018 [2, 7]. Food purchased from the OHFS tends to be energy dense [4, 3], and consumers often underestimate the energy content of food purchased from the OHFS [6, 7]. Frequently eating food from the OHFS is associated with poorer overall diet quality and obesity [1, 8].

In response to the growing contribution of the OHFS to diets, multiple countries, including the US [9] and parts of Canada [10] have implemented mandatory kcal labelling legislation. In 2011, the UK public health responsibility deal [11] was launched creating an industry and public health partnership with companies making voluntary pledges to improve public health. One of these pledges was for OHFS businesses in England to voluntarily provide kcal labelling [12]. Following this, a survey was conducted in 2018 examining kcal labelling practices in selected major eating out food chains in England including full-service and fast-food restaurants, cafes and coffee shops. It was found that only 18 out of 104 (17%) outlets assessed provided in-store labelling [13]. Furthermore, labelling was found to be inconsistent across businesses, often lacking prominence and clarity, and not being presented on all eligible menu items.

Motivated by a lack of voluntary compliance within the sector, government consulted on mandating kcal labelling in the OHFS in 2018 and following consultation [14], the UK government publicly announced in May 2021 to implement mandatory kcal labelling among large food businesses in England. This legislation came into force on 6th April 2022 [15]. The Kcal Labelling (Out of Home Sector) (England) Regulations 2021 [16] (the kcal labelling policy for short) requires large (250 or more employees) businesses (cafes, fast-food outlets, sit-down restaurants, pubs) in England selling food selling food and non-alcoholic drinks in scope of the Regulations to (1) display the energy content of the food in kcals (2) reference the size of the portion to which the kcal information relates (3) display the statement that ‘adults need around 2000 kcal a day’. Food and soft drinks are in scope of the regulations if they are: (1) offered for sale in a form which is suitable for immediate consumption, (2) not prepacked food and (3) not exempt food such as food on a temporary menu for less than 30 consecutive days [16]. The extent to which kcal labelling was implemented in anticipation of the policy or quality of existing labelling in eligible businesses prior to the out of home kcal labelling regulations has not been examined and will be of importance to interpreting the effectiveness of this policy when implemented.

Although research has examined the kcal content of food products sold in the OHFS in England [5, 17], there is a lack of data on the typical number of kcals purchased and consumed by customers in this sector. Moreover, research from other countries has indicated that out-of-home food consumption can vary based on socio-demographic factors such as gender [18, 19] and age [20–22]. Furthermore, obesity and poor-quality diet are associated with lower socioeconomic positions (SEP) [23, 24]. People of lower SEP are more likely to report being less motivated by weight management or healthiness of food when making dietary choices [25] and this may impact on behaviour in the OHFS. However, purchasing and consumption behaviour in England’s OHFS has not been examined in relation to socio-demographic factors. Investigating this is important prior to the policy implementation to assess whether the policy may reduce or widen existing health inequalities [26].

Additionally, the impact of mandatory kcal labelling on consumer behaviour in the OHFS is likely to be dependent on consumers’ level of engagement [27] and whether they notice and use kcal labelling when making food and drink selections [28]. Research from the US [29] and Canada [10] has found low rates of reported noticing (35–65%) and use (16–17%) of kcal labelling. Levels of obesity and consumer behaviour particularly in the OHFS vary between the US, Canada and England [30]–[31] and this highlights the needed to assess and examine kcal labelling practices and effects on consumer behaviour across countries. However, research has not investigated the proportion of consumers in England that report noticing and using kcal labelling when making purchases in the OHFS.

In the research reported here, OHFS outlets subject to the 2022 kcal labelling policy in England [32] were sampled in the year prior to its implementation (2021). The main objectives were to: (1) assess kcal labelling practices in the OHFS and how pre-implementation practice compares to previously reported data; (2) examine customer purchasing and consumption behaviour and kcal knowledge of their purchases; (3) assess customer noticing and use of kcal labelling in the OHFS; and (4) examine socio-demographic variations in consumer purchasing behaviour and kcal labelling use.

## Methods

### Study design

A field observation study of OHFS outlets in four areas of England was conducted.

### Outlet sampling

Four local authorities in England (Liverpool, Dudley, Milton Keynes, Richmond upon Thames) were selected in which to conduct this study. The local authorities were selected to ensure representation across quintiles of deprivation (measured for the purposes of selection of local authorities as Index of Multiple Deprivation (IMD) [33] at the local authority level) and geographical coverage across the South, North and Midlands and London areas of England. IMD levels 1–5 were used with IMD1 reflecting the most deprived areas and IMD5 representing the least deprived areas, defined at the lower layer super output area [34] (LSOA) to better capture small area geographic variations in IMD.

The Inter-Departmental Business Register [35] was used to identify businesses subject to mandatory kcal labelling policy (data sampled in June 2021, list produced in Autumn 2020). The Inter-Departmental Business Register is a list of UK businesses and their core characteristics including principal activities and the number of employees, used by the government for statistical purposes with the principal activities of businesses defined using Standard Industrial Classification [36]. Identified Standard Industrial Classification codes likely to include businesses serving food (for the full list of Standard Industrial Classification codes we used see supplementary materials) were first identified. Large businesses with 250 or more employees globally were then identified.

Outlets belonging to each identified large business (individual businesses could contribute multiple outlets, e.g. chain restaurants) within the four study areas using Ordnance Survey Points of Interest [37] data from September 2020 were identified. Outlets were sampled from each local authority and stratified by business type (restaurants; pubs and bars; retail; hotels; cafes; fast food; attractions and entertainment) and IMD quintile (1–5) according to categories provided by Ordnance Survey. We identified 902 outlets that were eligible within the four local authorities sampled (supplementary materials). After efforts to interview customers at hotels, attractions, and retail were unsuccessful, we removed these categories from eligibility for the customer intercept surveys, leaving 795 remaining eligible outlets for sampling (supplementary materials). From those 795 outlets that were eligible for sampling, we used stratified random sampling according to the IMD deprivation by business type proportions to identify 86 outlets per local authority in the first instance.

Any outlets found to be closed, not selling food subject to the mandatory kcal labelling policy, or who would not give permission for data collection on visiting were re-sampled and replaced with an outlet of the same business type and IMD deprivation level. If there were no remaining outlets local authority, the outlet was resampled from the nearest IMD deprivation level.

The data reported here are based on pre-policy data collection from a larger study examining kcal labelling practices and consumer behaviour pre and post the mandatory out of home kcal labelling regulations in England. See online supplementary materials for sample size calculation.

**Fig. 1:**
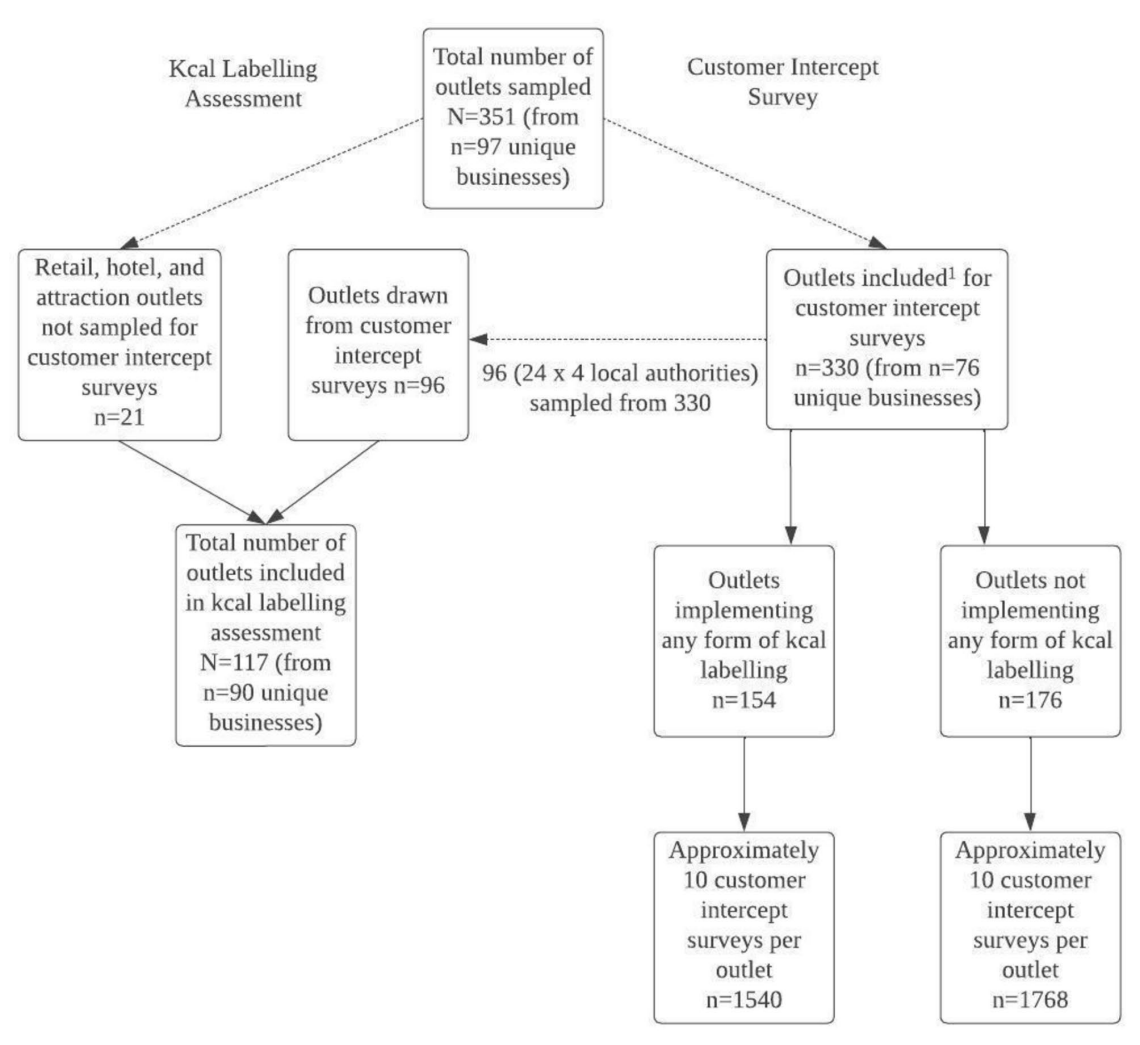
Sampling procedure for kcal labelling assessments and customer intercept surveys Note: Customer intercept surveys included cafes, fast-food, pubs, restaurants, and entertainment outlet types

In total, 351 outlets (330 outlets for customer intercept surveys and an additional 21 outlets that did not allow access to customers but were still checked for labelling compliance) were sampled across the kcal labelling assessments and customer intercept surveys.

### Customer intercept survey sampling

To examine customer purchasing and consumption behaviour, kcal knowledge of purchases, and noticing and use of kcal labelling in the OHFS, customer intercept surveys were attempted in all 351 outlets. Outlets requiring permission to collect data on their property were approached in advance via email, phone or instore contact, however permission was frequently withheld by store managers or area managers for data collection in retail, attraction, and hotel outlets, resulting in 21 outlets from these categories not being sampled for customer intercept surveys. The final sample for customer intercept surveys was 330 outlets (from 76 unique businesses) including cafes, fast-food, pubs, restaurants, and entertainment. See Fig. 1. Approximately 10 participants per outlet were recruited to complete the customer intercept survey resulting in a participant sample size of N = 3308.

### Kcal labelling assessment sampling

Kcal labelling practices were assessed in a subsample of outlets. Sampling outlets from unique business outlets (as opposed to sampling outlets from the same business) was prioritised to capture a wide range of kcal labelling practices across the OHFS. We aimed to sample the same number of outlets from each local authority (24 from each of the 4 local authorities). This resulted in 96 outlets being sampled and 69 were unique business outlets (due to limited availability of unique outlets in some local authorities). In addition, 21 outlets from the retail, attraction, and hotel outlet types that were unable to be included for the customer intercept surveys due to a lack of permission were included in this sample. The final sample for kcal labelling assessments was 117 outlets (from 90 unique businesses) across the four study local authorities including outlets from attractions, cafes, fast-food, hotels, pubs, restaurants, retail and sport and entertainment businesses (full list of chains in supplementary materials). See Fig. 1. For the kcal labelling assessments, (N = 117 outlets) results were reported descriptively.

### Customer intercept survey procedure

Intercept surveys with customers were conducted to examine purchasing, consumption, kcal knowledge and noticing and use of kcal labelling. To be eligible for inclusion participants were required to have purchased at least one food item from the selected outlet and be aged 16 or over. All participants provided informed verbal consent and ethical approval was granted by the University of Liverpool’s Ethics Committee.

Researchers stood outside the selected food outlets during peak operating hours for a midday meal and evening meal (typically 12 pm – 9 pm, Wednesday to Sunday) and recruited customers when entering or exiting the outlet. Participants completed a short exit survey after consuming their food and drink purchases (survey questions in supplementary materials). Basic demographic information was collected (age, gender, ethnicity, and highest education level) with education level used to indicate participants’ SEP (lower SEP = school leaving, A-level or equivalent qualifications or lower and higher SEP = qualifications above school leaving A-levels or equivalent). Questions were asked about whether participants noticed kcal labelling in the outlet (yes/no), whether they used this when making their purchases (yes/no) and if yes, why (to select lower-kcal options, to select higher kcal options, other) and how (selected alternative meal option, selected smaller/larger portion, made a meal substitution or customisation).

To assess participants’ kcal knowledge, participants estimated the total number of kcals in their purchases. Participants were asked to report all food and drink purchased from the outlet for their own consumption and asked to indicate any instances where items were shared and, if so, what proportion was shared, and estimate what proportion, if any, was left uneaten. Reports of shared items and leftover estimates were used to calculate consumption values for each participant. Where possible, customers were asked to share receipts to verify purchases – although many outlets were not issuing receipts during data collection due to hygiene concerns related to the covid-19 pandemic. Participants were informed that the study was investigating dining habits to minimise influencing participants’ purchasing behaviour and avoid increasing their focus on kcal labelling. All methods were carried out in accordance with relevant guidelines and regulations.

### Kcal labelling assessment procedure

To examine kcal labelling practices, a method based on that of Robinson et al. [13] was used. Briefly, researchers visited selected outlets to assess whether any kcal labelling was present and, if so, whether it adhered to the best practice recommendations for kcal labelling provided by the Department of Health and Social Care [38] (Table 1). Kcal labelling was rated for adherence according to the following criteria: (1) kcal labelling is provided at any point of choice (2) kcal labelling is provided at all points of choice, (3) kcal labelling is provided for eligible food items, (4) kcal labelling is provided per portion for sharing items, (5) kcal labelling is presented close to the item’s name and price, (6) kcal labelling is presented as prominently as name or price, (7) kcal reference information is displayed, (8) kcal reference information is displayed clearly and prominently (9) kcal labelling is provided for all non-alcoholic drink items. Researchers examined handheld food and drink menus, point-of-choice menu display boards inside and outside the outlet and menus presented at ordering points. Researchers rated adherence with kcal labelling best practice recommendations (0 = no, 1 = yes) and a score between 0 and 9 was calculated for each outlet based on the number of kcal labelling practices the outlet adhered to. For example, a score of 3 would mean that the outlet adhered to 3 of the best practice recommendations (list of criteria in Table 1 and supplementary materials). For each Researchers received training on adherence to best practice recommendations for kcal labelling and the study protocol prior to the start of data collection to ensure consistency between raters. 10% (n = 17) of outlets were coded by a second researcher with a percentage agreement score of 96% across all 9 variables.

**Table 1:** Outlet Characteristics (Outlet type, local authority and LSOA IMD value) for kcal labelling assessments and customer intercept surveys

	Outlets included in kcal labelling assessments N = 117	Outlets included for customer intercept surveys N = 330
Local Authorities N (%)		
Liverpool	30 (26%)	86 (26%)
Dudley	28 (24%)	84 (25%)
Milton Keynes	30 (26%)	82 (25%)
Richmond	29 (24%)	78 (24%)
Outlet Type N (%)		
Attractions	3 (3%)	0 (0%)
Cafes	10 (9%)	66 (20%)
Fast-food	11 (9%)	81 (25%)
Hotels	13 (11%)	0 (0%)
Pubs	26 (22%)	92 (27%)
Restaurants	43 (37%)	81 (25%)
Retail	5 (4%)	0 (0%)
Entertainment	6 (5%)	10 (3%)
LSOA IMD Quintiles N (%)		
1	39 (33%)	94 (29%)
2	16 (14%)	47 (14%)
3	21 (18%)	59 (18%)
4	9 (8%)	40 (12%)
5	32 (27%)	90 (27%)

*Note: IMD = Indices of Multiple Deprivation*

### Extraction of meal kcal content

Information from an existing nutritional database (MenuTracker) [39] was used to calculate the total kcal content of each participant’s food and drink purchases and the amount consumed. MenuTracker contains web scraped nutritional information for menu items on the websites of large UK OHFS businesses. Data is collected quarterly and data from the September 2021 scrape was used to calculate kcal content. In instances where the kcal content was not available from MenuTracker, nutritional content was sought from the business’s website. In instances that multiple menu item options were available on the database, or the item was not identifiable (for example, if it was unclear which menu item was purchased), one of the following solutions was applied in order of viability: (1) closest item matched from the database; (2) mean taken of multiple menu items that could be a possible match; (3) item coded as missing.

### Data exclusions

Outlets that did not have kcal information available in either MenuTracker or via a separate web search were excluded from analyses assessing outlet characteristics (Outlet type, deprivation level and local authority) on the number of kcals purchased and consumed and kcal estimates. Participants were excluded from the kcals purchased, kcals consumed, and kcal estimates analysis if the total number of kcals they purchased was unavailable or incomplete (although these participants were retained in the noticing and use of kcal labelling analyses). Numbers of exclusions are reported by reason for missing data in supplementary materials.

### Data analysis

To determine whether participant demographics were associated with purchasing behaviour, data from the customer intercept surveys were analysed using linear regression models with cluster robust standard errors, with individual outlets used as the cluster variable. R and R Studio version 1.2.503 was used to conduct analyses. For the linear models, age, gender, ethnicity, SEP, outlet type, IMD value, local authority and kcal labelling presence were planned exposure variables. The number of kcals purchased, consumption (adjusting for leftover estimates and shared items), and customer kcal estimates were outcomes. The day of the week the outlet was visited (weekday or weekend) and time of day (midday meal 12 − 4 pm or evening meal 5–9 pm) were additional covariates in these models to control for potential variations in customer eating behaviours based on the time/date of the visit to control for variations in eating habits. As the energy content of meals may influence the accuracy of kcal estimates, models examining kcal estimates also included total kcals purchased to control for meal kcal content. During initial analyses it was noted there was substantial collinearity between local authority and IMD (VIFs > 8), so we opted to retain IMD within the models only. Models with local authority, rather than IMD are reported in supplementary materials. Models with only the demographic characteristics as exposure variables (age, gender, ethnicity, and SEP) are reported in the supplementary materials.

Logistic regression models were used to assess whether demographics or outlet characteristics were associated with the odds of customers using or noticing kcal labelling. Exposure variables were outlet characteristics (outlet type, LSOA IMD, whether kcal labelling was present in outlet) and participant demographics (age, gender, ethnicity, and SEP). Additional covariates included were weekend vs. weekday and midday vs. evening meal. To calculate the difference in kcal estimates, the number of kcals purchased was subtracted from participants’ kcal estimations, meaning positive numbers represent an overestimation of kcals purchased, and negative numbers represent an underestimation. To account for the relatively large number of analyses conducted 99% confidence intervals are reported to determine statistical significance.

## Results

Outlet characteristics (outlet type, local authority and LSOA IMD value) for the included outlets are reported in Table 2.

**Table 2:** Frequencies of kcal labelling implemented in the outlets

	Total outlets (117) N (%)
Is kcal labelling provided at any point of choice?	24 (21%)
Is kcal labelling provided at all points of choice?	16 (14%)
Is kcal labelling provided for all food items?	17 (15%)
Is kcal labelling provided per portion for sharing menu items?	10 (9%)
Is kcal labelling presented close to the item’s name and price?	21 (18%)
Is kcal labelling presented as prominently as name or price?	0 (0%)
Is kcal reference information displayed anywhere?	11 (9%)
Is kcal reference information displayed clearly and prominently?	4 (3%)
Is kcal labelling provided for all non-alcoholic drink items?	11 (9%)

Note: Total outlets = 117 individual outlets from across 90 unique businesses

### Kcal labelling assessment

Table 1 shows 24 outlets from 117 examined (21%) had any instance of kcal labelling, with a mean labelling score of 4.8/9 in these 24 outlets (calculated based on criteria in Table 1). Frequencies of the nine kcal labelling criteria are shown in Table 1. Of the 24 outlets providing labelling, 6 were cafes (Mean labelling score = 3.7/9), 6 were fast food (Mean labelling score = 6.2/9), 5 were pubs (Mean labelling score = 4.4/9), 2 were restaurants (Mean labelling score = 3.0/9), 4 were retail (Mean labelling score = 6.3/9) and 1 was a hotel (Mean labelling score = 2.0/9). The attraction and entertainment venues (i.e., cinemas, zoos, sports venues) sampled did not provide labelling (Mean labelling score = 0/9).

### Customer Intercept Survey

Descriptive information about the participants and outlet types is shown in Table 3.

**Table 3:** Participant demographics (Age, Gender, Ethnicity and SEP) and outlet types

	Participant Demographics (N = 3308)
Age (mean(SD)) Age Range (years)	41.0 (18.7) 16–92
Gender (M: F)	1682:1622
SEP High	1191 (36%)
SEP Low	2117 (64%)
White	2787 (84%)
Asian	208 (6%)
Black	152 (5%)
Mixed Race	85 (3%)
Other	56 (2%)
***Participants per outlet type***	
Cafes	654 (20%)
Entertainment	101 (3%)
Fast Food	813 (24%)
Restaurant	819 (25%)
Pub	921 (28%)

*Note: SEP = Social economic position. Low SEP indicated school leaving, A-level or equivalent qualifications or lower, High SEP indicates qualifications above school leaving A-levels or equivalent*

### Kcals purchased

Across the N = 3308 participants recruited from N = 330 outlets, the mean number of kcals purchased was 1013 (SD = 632) with 69% of purchases exceeding 600 kcals (recommended number of kcals for a meal in the UK [40]. There was considerable clustering of kcals purchased within individual outlets (ICC = 0.50). For the model consisting of demographic information, outlet characteristics and covariates, age (younger adults purchased more) gender (males purchased more), and whether it was a weekend vs. weekday (more kcals purchased at weekends) were significant predictors of kcals purchased. For outlet characteristics, compared to the reference category of cafes, entertainment venues, fast food venues, pubs and restaurants were all associated with increased kcal purchasing. Outlets located in IMD5 compared to IMD1 were associated with decreased kcal purchasing (Table 4). The model consisting of only demographic information and covariates is reported in the supplementary materials.

**Table 4:** Demographic and outlet predictors of kcals purchased, consumed and kcal estimates

	Kcals Purchased Demographic and Outlet Model [1] B [99% CI]	Kcals Consumed Demographic and Outlet Model B [99% CI]	Kcal estimates Demographic and Outlet Model B [99% CI]
Age	-2.184 [-3.815; -0.554] *	-1.83 [-3.33; -0.33] *	1.40 [-0.09; 2.82]
Male (v. Female)	75 [18; 132] *	111 [58; 164] *	58 [10; 106] *
Non-White (v. White)	-71 [-150; 8]	-54 [-128; 19]	-51 [-118; 17]
Low SEP (v. High)	8 [-53; 69]	29 [-28; 86]	-71 [-135; -6] *
Midday (v. Evening)	-106 [-221; 9]	-73 [-171; 25]	-87 [-178; 5]
Weekend (v. Weekday)	109 [8; 210] *	69 [-19; 157]	20 [-50; 89]
Entertainment (v. Cafe)	227 [-1; 456] *	-6 [-197; 184]	-103 [-226; 20]
Fast Food (v Cafe)	274 [178; 371] *	214 [128; 299] *	187 [117; 258] *
Pub (v. Cafe)	958 [804; 1111] *	867 [732; 1002] *	61 [-53; 175]
Restaurant (v. Cafe)	743 [587; 899] *	629 [487; 770] *	357 [283; 475] *
IMD2 (v.IMD1)	-50 [-206; 106]	-61 [-190; 67]	1 [-98; 100]
IMD3 (v.IMD1)	-68 [-196; 60]	-30 [-155; 95]	-2 [-75; 70]
IMD4 (v.IMD1)	-67 [-196; 61]	-59 [-172; 53]	11 [-101; 123]
IMD5 (v.IMD1)	-129 [-248; -9] *	-98 [-204; 9]	-13 [-107; 81]
Labelling Present	30 [-97; 158]	6 [-101; 112]	32 [-46; -111]
Kcals Purchased	-	-	-0.74 [-0.65; -0.83]*
Num. obs.	2446	2446	2440
N Clusters	289	289	289

^*1*^ *Note: Models include age, gender, ethnicity, SEP, outlet type, LSOA IMD value, local authority, and kcal labelling presence as exposure variables. Models examining kcal estimates included total kcals purchased to control for meal kcal content. Day of the week (weekend vs. weekday) and time of day (midday or evening) were additional covariates in these models*

Table legend: Reference categories (females, white, high SEP, Evening meal, Weekday, Cafes, IMD1 and labelling absent). IMD = Indices of Multiple Deprivation, SEP = Social economic position. IMD1 represents the most deprived areas of the UK and IMD5 represents the least deprived areas. In relation to kcal estimates, positive values represent an overestimation and negative values represent an underestimation of kcal content

### Kcals consumed

Across the N = 3308 participants recruited from N = 330 outlets, participants consumed 915 kcals (SD = 578) on average. There was clustering of consumption within individual outlets (ICC = 0.49). For the model consisting of demographic information, outlet characteristics and covariates, age (younger adults consumed more) and gender (males consumed more), were significant predictors of consumption. Compared to the reference category of cafes, fast food venues, pubs, and restaurants were all associated with increased consumption. There was no association with IMD (Table 4). The model consisting of only demographic information and covariates is reported in the supplementary materials.

### Participant kcal estimates

Participants underestimated the number of kcals in their purchased meal by on average of 253 kcals (SD = 644 kcals). For the model consisting of demographic information, outlet characteristics and covariates, kcals purchased (greater underestimation for higher kcal purchases), gender (greater underestimation for males) and SEP (Low SEP had greater underestimation) were significant predictors of kcal estimates (Table 4). Compared to the reference category of cafes, there was a significant underestimation of kcals in both fast-food outlets and restaurants.

### Noticing and use of kcal labelling

Analyses of noticing and using kcal labelling reported in customer intercept surveys were limited to participants who were recruited from outlets with the presence of kcal labelling. This was determined based on the kcal labelling assessment sampling and if a sampled outlet from a multi-outlet business provided kcal labelling, it was assumed this was the case for other outlets of that business (N outlets with presence of kcal labelling = 154/330, N participants = 1540). Overall, N = 324 (21%) participants reported noticing kcal labels. For the demographic and outlet model, the only significant predictors were gender (women noticed labelling more than males), and the outlet being a pub (v. cafe). Full results are presented in supplementary materials.

Of the 324 participants who reported noticing kcal labels, 65 (20%) reported using kcal labels to make purchase decisions. This means that of n = 1540 potentially exposed to kcal labelling, only 4% reported it made an impact on their purchasing decision. The majority of these (> 95%) reported they ordered a lower number of kcals as a result of labelling.

## Discussion

### Summary of main findings

This study examined kcal labelling practices, customer purchasing behaviour and levels of noticing and using kcal labelling in the OHFS during 2021, after the announcement of, but prior to the kcal labelling policy coming into force in England. It was found that a minority of sampled businesses provided any form of kcal labelling (21% of 117 largely unique business outlets) at this time, and when present, labelling was never fully reflective of all best practice recommendations for kcal labelling in the OHFS. The mean energy content of purchases per customer (1013 kcal) and energy consumed (915 kcals) were both high compared to UK public health recommendations of 600 kcals per meal [40] (69% of purchases exceeded 600kcals). Customers tended to underestimate the energy content of their purchases by an average of 253 kcals. In outlets that did provide kcal labelling, customer’s reported noticing (21%) and use amongst those noticing (20%) kcal labelling was low meaning that only 4% of those potentially exposed to kcal labelling reported that it impacted their purchasing decision.

### Relationship to previous knowledge and implications

The energy content of purchased and consumed meals is broadly consistent with previous research indicating that food purchased in the OHFS is high in kcals and likely to be contributing to population level energy overconsumption [4, 5]. In a 2018 study [13], 17% of sampled businesses in England provided any form of kcal labelling, compared to 21% in the present study, indicating similar results and that there was not a substantial improvement in kcal labelling practices in the OHFS since the 2018 assessment [13]. However, it should be noted that these studies examined different samples of major OHFS chains and therefore are not directly comparable. Mandatory kcal labelling in the OHFS could lead to reductions in kcal consumption in two ways; by influencing individuals’ choices [9] and through menu reformulation [41]. Future research examining whether mandatory implementation of kcal labelling changes consumer behaviour (e.g., utilising purchase data) or reformulation (e.g., utilising product nutrition information) will be informative. Increased levels of implementation, as well as ensuring this is done according to recommendations to maximise the likelihood of customers noticing it, as a result of mandatory kcal labelling policy, could help mitigate the extent to which the OHFS contributes to overconsumption. Wider implementation, more consistent, and prominent labelling may increase customers noticing, and in turn use of kcal labelling, potentially leading to a greater awareness of kcal content of meals and the selection of lower kcal menu options. Participant’s potential lack of understanding kcal labelling may be a barrier to using in-store kcal labelling. Previous research conducted in the US found that only 64–73% of the public were able to accurately report daily kcal needs [42]. Due to this, public education campaigns may be required, focusing on kcal requirements to aid understanding or additional labelling formats that define foods as “low”, “moderate” or “high” kcal. However, research conducted in the UK examining supermarket food labels found that understanding was relatively high with up to 87.5% of respondents able to identify the healthiest product [43]. Therefore, future research is needed to examine the full extent of people’s understanding of kcal labelling in the UK OHFS and whether education campaigns or additional support is needed to promote greater use of kcal labels.

Furthermore, similar to the effect of front-of-package product nutritional labels [44, 45], mandatory kcal labelling policy may lead to reformulation resulting in a potential reduction in kcal content of menu items [17]. Studies have found a reduction in kcal content of prepared supermarket food following the implementation of kcal labels, suggesting that menu reformulation (as opposed to consumer choice) could lead to overall reductions in kcal intake [41]. It is possible that the implementation of kcal labelling could result in a similar reduction in kcal content of existing menu items and potentially increased availability of lower kcal menu options for consumers [46].

Companies may sometimes align their business practices with upcoming regulations ahead of their implementation [47], however this did not appear to be the case in the current study. This may be due to barriers identified by companies such as time constraints [48]. Furthermore, prior to the announcement of the kcal labelling policy companies reported that implementing kcal labelling was of low priority [48]. Additionally, due to the time-period examined in this study (3 months following the policy announcement and 8 months prior to the implementation date), many companies may have been in the process of preparing to implement in-store kcal labelling.

Previous research has indicated sociodemographic variations in purchasing and consumption behaviour in the OHFS and this was also observed in the present study. As previously noted, males purchased and consumed meals with a higher energy content than females [18, 19]. In the present study, there was a significant difference in the amount of energy purchased and consumed based on age. This finding corresponds with data from the UK, Netherlands and Ireland indicating that young adults (16–25 years old) purchase and consume higher kcals in the OHFS compared to older age groups [20–22]. In the present study there was no significant difference in the amount of energy purchased or consumed based on SEP or ethnicity. Little research has directly examined purchasing and consumption variations in the OHFS based on ethnicity, however, a US study reported that being from a Hispanic background and lower SEP related to eating more meals away from home, as opposed to being white or from a higher SEP background [49]. Due to the nature of this study, SEP was characterised based on the participant’s highest education as opposed to other more sensitive methods such as household income. However, it may be the case that SEP measures relating to household income or wealth would be associated with the energy content of purchases. Energy-dense foods are often cheaper to produce, and this may result in an association with income level. A review found that foods of lower nutritional value generally cost less per kcal and were more frequently selected by groups from lower SEP [50]. Related to this, it was found that there were fewer kcals purchased from outlets in the least (IMD5) versus most deprived areas (IMD1). Previous studies have found a higher prevalence of OHFS outlets in more deprived areas [51], potentially creating greater businesses competition which results in decreased price, larger portion sizes and/or lower quality ingredients.

Consistent with previous studies, participants in the present research tended to underestimate the energy content of meals purchased in the OHFS [52, 5]. Previous research has shown greater underestimation of energy content for higher kcal meals [7] and this was observed in the present study. Greater underestimation may have been caused by the energy density and/or portion size of meals higher in energy, as previous research indicates that larger portion sizes tend to be prone to greater underestimation than smaller portion sizes of food [53]. People with lower SEP also showed greater underestimation of energy content of purchased meals. This may be due to variations in education level and knowledge of the energy content of food items as research has indicated that US adults with higher income and education levels are more likely to accurately estimate typical daily kcal needs [54].

Multiple studies have examined customers’ levels of noticing and use of kcal labelling, and variations in these based on demographic factors. Here, 21% of participants reported noticing kcal labels and only 20% of those who noticed them reported using them when making their purchases (4% across total sample). This is a relatively low figure compared to figures from the US (60% noticed kcal labelling and 16% reported using kcal labelling) [29] and Canada (35% noticed kcal labelling and 17% reported being influenced by it) [10]. However, it should be noted that these figures for the US and Canada were taken post-implementation of mandatory kcal labelling, and levels of labelling awareness may increase in England post-implementation of mandatory kcal labelling. Research has found higher reported noticing of kcal labelling after the implementation of mandatory kcal labelling compared with voluntary policies [10]. The present findings suggest that kcal labelling has not been widely implemented or prominently presented, likely contributing to the low levels of awareness and use of kcal labelling in England. Although the implementation of mandatory kcal labelling does not equate to use, increased availability, consistency and prominence of labelling, that often accompanies the implementation of mandatory labelling policy, may lead to increased noticing and use. Implementation of the mandatory kcal labelling policy in April 2022 may lead to an increase in awareness, use and knowledge of kcal content in OHFS meals, however future research is required to determine the effects of mandatory kcal labelling in the UK OHFS on consumer behaviour and wider public health. Given the relatively low levels of noticing and use of kcal labelling in businesses providing labelling in the present study, it is recommended that future implementations of similar policies ensure that labelling is presented clearly and prominently (e.g., similar size as price or name of menu items) to maximise noticing and use. Future research examining whether variations in the presentation of kcal labelling such as colour, text size or use of traffic light labels [55] could help improve participants noticing, use and understanding of kcal labels may help inform policy and best practice recommendations for kcal labelling in the OHFS.

### Strengths and weaknesses

This is the first study to our knowledge that has examined real-world OHFS purchasing, consumption and noticing of kcal labelling in England. The study recruited a large number of participants and food outlets from local authorities sampled from each economic deprivation level in England.

A limitation of the study is the amount of missing data due to nutritional information not being made available by some OHFS outlets (25% of sampled outlets did not have kcal information available). It is not clear whether the results examining energy content of customer purchases and consumption data would differ if these outlets were included in the analyses. For example, outlets with higher kcal items may be more reluctant to publish nutritional content. Furthermore, although this study included outlets from a range of deprivation levels and various parts of England (South, North and Midlands and London areas) it can not be assumed that the data collected is representative of all areas of England. A further limitation is that at the participant level this study only measured education level to define SEP. Although this study also measured area-level SEP and this in part accounts for local area wealth [56], it would be informative for future research to measure participant-level income as this may relate to kcal labelling usage.

Two factors could lead to inaccuracies and potential underestimations in the data presented for kcals purchased and consumed: (1) inaccuracies in customer reporting and (2) inaccuracies in nutritional information from food outlets. Therefore, a limitation of this study is the reliance on self-reporting of food purchases and the amount consumed. Objectively measured kcal content, such as using laboratory measurements to weigh leftovers and analyse kcal content, would have been preferable but was not feasible in this study. However, to mitigate the potential for inaccurate reporting, food purchases were recorded shortly after consumption and where possible customer receipts were used to verify purchases. Previous research has also indicated that commercially provided nutritional information is, on the whole, accurate but may underestimate the kcal content of some food items [57, 58]. As a result, the findings presented here are likely to be an underestimation rather than an overestimation of purchasing and consumption behaviour in the OHFS.

There are a wide range of outlets in scope of the new mandatory kcal labelling policy in England some of which we were unable to include for our customer intercept surveys, such as hotels, attractions (e.g., cinemas) and food delivery outlets. As a result, this study is unable to provide a full representation of consumer behaviour across all aspects of the OHFS subject to kcal labelling policy in England. A final limitation is that for a small percentage of participants aged 16–18 years old, some may have been studying and shortly achieving a higher educational qualification, but our classification for higher SEP (highest education qualification achieved: A-levels or equivalent) would result in them being categorised as lower SEP.

### Conclusions and future research

The number of OHFS outlets providing kcal labelling around 6 months prior to the Kcal Labelling (Out of Home Sector) (England) Regulations 2021 coming into force was low. When labelling was provided it was done so inconsistently, lacking prominence and clarity. Customer noticing and use of labelling was low. The average number of kcals purchased and consumed in the OHFS was substantially more than recommended by public health guidelines. Furthermore, customers often underestimated the energy content of their purchases. Future research should assess whether kcal labelling reduces the number of kcals purchased and the level of noticing and use of kcal labelling in the OHFS to examine the impact of the 2022 kcal labelling policy in England.

## Electronic supplementary material

Below is the link to the electronic supplementary material.

Supplementary Material 1

## Declarations

### Data sharing

Due to research ethics approval permission, data from customer intercept surveys and kcal labelling assessment data is available on request from the authors from January 2023 onwards.

## Abbreviations

DHSC: Department of Health and Social Care
ICC: Intraclass Correlation Coefficient
IMD: Indices of Multiple Deprivation
KCAL: Kilocalorie
LSOA: Lower layer Super Output Area
OHFS: Out-of-home food sector
SEP: Socioeconomic Position
VIF: Variance Inflation Factor

## References

[1] World Health Organization. The out-of-home food environment: report of a WHO Regional Office for Europe and Public Health England expert meeting, 10 June 2021. World Health Organization. Regional Office for Europe; 2022.

[2] Adams J, Goffe L, Brown T, Lake AA, Summerbell C, White M, Wrieden W, Adamson AJ (2015). Frequency and socio-demographic correlates of eating meals out and take-away meals at home: cross-sectional analysis of the UK national diet and nutrition survey, waves 1–4 (2008–12). Int J Behav Nutr Phys Act.

[3] Mills S, Adams J, Wrieden W, White M, Brown H. Sociodemographic characteristics and frequency of consuming home-cooked meals and meals from out-of-home sources: cross-sectional analysis of a population-based cohort study. Public Health Nutr. 2018;21(12):2255–66. 10.1017/S1368980018000812 PMC6064641 29637874

[4] Roberts SB, Das SK, Suen VM, Pihlajamäki J, Kuriyan R, Steiner-Asiedu M, Taetzsch A, Anderson AK, Silver RE, Barger K, Krauss A. Measured energy content of frequently purchased restaurant meals: multi-country cross sectional study. BMJ. 2018;363. 10.1136/bmj.k4864 PMC6290458 30541752

[5] Robinson E, Jones A, Whitelock V, Mead BR, Haynes A. (Over) eating out at major UK restaurant chains: observational study of energy content of main meals. BMJ. 2018;363. 10.1136/bmj.k4982 PMC6290483 30541906

[6] Elbel B (2011). Consumer estimation of recommended and actual calories at fast food restaurants. Obes (Silver Spring Md).

[7] Block JP, Condon SK, Kleinman K, Mullen J, Linakis S, Rifas-Shiman S, Gillman MW (2013). Consumers’ estimation of calorie content at fast food restaurants: cross sectional observational study. BMJ: Br Med J.

[8] Bezerra IN, Curioni C, Sichieri R. Association between eating out of home and body weight. Nutrition reviews. 2012;70(2):65–79. 10.1111/j.1753-4887.2011.00459.x 22300594

[9] Cleveland LP, Simon D, Block JP. Compliance in 2017 with federal calorie labeling in 90 chain restaurants and 10 retail food outlets prior to required implementation. Am J Public Health. 2018;108(8):1099–102. 10.2105/AJPH.2018.304513 PMC6050842 29927646

[10] Goodman S, Vanderlee L, White CM, Hammond D. A quasi-experimental study of a mandatory calorie-labelling policy in restaurants: impact on use of nutrition information among youth and young adults in Canada. Prev Med. 2018;1:116:166–72. 10.1016/j.ypmed.2018.09.013 30261242

[11] Department of Health. & Social Care. Public health responsibility deal. 2011.

[12] Jebb SA (2011). Calorie labelling on the high street. BMJ (Clinical research ed).

[13] Robinson E, Burton S, Gough T, Jones A, Haynes A. Point of choice kilocalorie labelling in the UK eating out of home sector: a descriptive study of major chains. BMC Public Health. 2019;19(1):1–6. 10.1186/s12889-019-7017-5 PMC6540449 31138179

[14] Department of Health & Social Care. Mandating calorie labelling in the out-of-home sector. Government response to public consultation. 2020. Mandating calorie labelling in the out-of-home sector: government response to public consultation (publishing.service.gov.uk).

[15] Department of Health & Social Care. Impact assessment: mandatory calorie labelling in the out-of-home sector. 2020. https://assets.publishing.service.gov.uk/government/uploads/system/uploads/attachment_data/file/992872/calorie-labelling-impact-assessment.pdf.

[16] Department of Health & Social Care. The Calorie Labelling (Out of Home Sector) (England) Regulations 2021. 2021. The Calorie Labelling (Out of Home Sector) (England) Regulations 2021 (legislation.gov.uk).

[17] Theis DR, Adams J. Differences in energy and nutritional content of menu items served by popular UK chain restaurants with versus without voluntary menu labelling: a cross-sectional study. PLoS One 2019;14(10):e0222773. 10.1371/journal.pone.0222773 PMC6795485 31618202

[18] Orfanos P, Naska A, Trichopoulos D, Slimani N, Ferrari P, van Bakel M (2007). Eating out of home and its correlates in 10 european countries. The european prospective investigation into Cancer and Nutrition (EPIC) study. Public Health Nutr.

[19] Vandevijvere S, Lachat C, Kolsteren P, Van Oyen H (2009). Eating out of home in Belgium: current situation and policy implications. Br J Nutr.

[20] Burke SJ, McCarthy SN, O’Neill JL, Hannon EM, Kiely M, Flynn A, Gibney MJ. An examination of the influence of eating location on the diets of irish children. Public Health Nutr. 2007;10(6):599–607. 10.1017/S1368980007258379 17381926

[21] Kearney JM, Hulshof KF, Gibney MJ (2001). Eating patterns–temporal distribution, converging and diverging foods, meals eaten inside and outside of the home–implications for developing FBDG. Public Health Nutr.

[22] O’Dwyer NA, Gibney MJ, Burke SJ, McCarthy SN (2005). The influence of eating location on nutrient intakes in irish adults: implications for developing food-based dietary guidelines. Public Health Nutr.

[23] Adams J (2020). Addressing socioeconomic inequalities in obesity: democratising access to resources for achieving and maintaining a healthy weight. PLoS Med.

[24] De Mestral C, Chatelan A, Marques-Vidal P, Stringhini S, Bochud M (2019). The contribution of Diet Quality to socioeconomic inequalities in obesity: a Population-based study of swiss adults. Nutrients.

[25] Robinson E, Jones A, Marty L. The role of health-based food choice motives in explaining the relationship between lower socioeconomic position and higher BMI in UK and US adults. Int J Obes. 2022;21:1–7. 10.1038/s41366-022-01190-4 PMC7613617 35864310

[26] Adams J, Mytton O, White M, Monsivais P (2016). Why are some Population Interventions for Diet and obesity more Equitable and Effective Than others? The role of Individual Agency. PLoS Med.

[27] Polden M, Robinson E, Jones A. Assessing public perception and awareness of UK mandatory calorie labelling in the out-of-home sector: using Twitter and Google trends data [Preprint]. arXiv [Preprint]. 2022. Available from: 10.31234/osf.io/t6bhy. 10.1002/osp4.674 PMC10551121 37810520

[28] Nikolaou CK, Hankey CR, Lean ME. Calorie-labelling: does it impact on calorie purchase in catering outlets and the views of young adults? Int J Obes. 2015;39(3):542–5. 10.1038/ijo.2014.162 25174452

[29] Green JE, Brown AG, Ohri-Vachaspati P. Sociodemographic disparities among fast-food restaurant customers who notice and use calorie menu labels. Journal of the Academy of Nutrition and Dietetics. 2015;115(7):1093 – 101. 10.1016/j.jand.2014.12.004 25659920

[30] Nieto C, Jáuregui A, Contreras-Manzano A, Potvin Kent M, Sacks G, White CM, Pauzé E, Vanderlee L, Thrasher JF, Barquera S, Hammond D. Adults’ exposure to unhealthy food and Beverage Marketing: a multi-country study in Australia, Canada, Mexico, the United Kingdom, and the United States. J Nutr. 2022;152(Supplement1):25S–34S. 10.1093/jn/nxab449 PMC9188858 35544288

[31] Contreras-Manzano A, Nieto C, Jáuregui A, Pérez Ferrer C, Vanderlee L, Barquera S, Sacks G, Adams J, Thrasher JF, Hammond D. Perceived availability of Healthy and Unhealthy Foods in the Community, Work, and higher education settings across five countries: findings from the International Food Policy Study 2018. J Nutr. 2022;152(Supplement1):47S–56S. 10.1093/jn/nxac070 PMC9188857 35544236

[32] Department of Health & Social Care. Mandating calorie labelling in the out-of-home sector.2020. https://assets.publishing.service.gov.uk/government/uploads/system/uploads/attachment_data/file/903714/Calorie_Labelling_-_Consultation_Response.pdf.

[33] National Statistics. English indices of deprivation. 2019. https://www.gov.uk/government/statistics/english-indices-of-deprivation-2019.

[34] Office for National Statistics. Rural urban classification (2011) of lower layer super output areas in England and Wales. 2011.

[35] Evans P, Welpton R (2009). Business structure database: the Inter-Departmental Business Register (IDBR) for research. Economic & Labour Market Review.

[36] Hughes JC, James G, Evans A, Prestwood D. Implementation of Standard Industrial Classification 2007: December 2009 update. Economic & Labour Market Review. 2009;3:51 – 5.

[37] Points of interest: technical information. https://beta.ordnancesurvey.co.uk/products/points-of-interest.

[38] Department of Health & Social Care. A Quick Guide to the Government’s Healthy Eating Recommendations. 2018. https://assets.publishing.service.gov.uk/government/uploads/system/uploads/attachment_data/file/742746/A_quick_guide_to_govt_healthy_eating_update.pdf.

[39] Huang Y, Burgoine T, Essman M, Thies D, Adams J. (Under review) MenuTracker Database: monitoring the nutrient composition of food prepared out-of-home in the UK; JMIR Public Health & Surveillance 10.2196/39033 PMC9501650 36074559

[40] Public Health England. Calorie reduction: the scope and ambition. 2018. https://www.gov.uk/government/publications/calorie-reduction-the-scope-and-ambition-for-action.

[41] Grummon AH, Petimar J, Soto MJ, Bleich SN, Simon D, Cleveland LP, Rao A, Block JP. Changes in calorie content of menu items at large chain restaurants after implementation of calorie labels. JAMA network open. 2021;4(12):e2141353-. 10.1001/jamanetworkopen.2021.41353 PMC8719240 34967879

[42] Krukowski RA, Harvey-Berino J, Kolodinsky J, Narsana RT, DeSisto TP. Consumers may not use or understand calorie labeling in restaurants. J Am Diet Assoc. 2006;106(1):917–20. 10.1016/j.jada.2006.03.005 16720133

[43] Grunert KG, Wills JM, Fernández-Celemín L. Nutrition knowledge, and use and understanding of nutrition information on food labels among consumers in the UK. Appetite. 2010;55(2):177 – 89. 10.1016/j.appet.2010.05.045 20546813

[44] Roberto CA, Ng SW, Ganderats-Fuentes M, Hammond D, Barquera S, Jauregui A, Taillie LS. The influence of front-of-package nutrition labeling on consumer behavior and product reformulation. Annu Rev Nutr. 2021;11:41:529–50. 10.1146/annurev-nutr-111120-094932 34339293

[45] Bleich SN, Wolfson JA, Jarlenski MP, Block JP. Restaurants with calories displayed on menus had lower calorie counts compared to restaurants without such labels. Health Affairs. 2015;34(11):1877-84. 10.1377/hlthaff.2015.0512 PMC5103638 26526245

[46] Robinson E, Marty L, Jones A, White M, Smith R, Adams J. Will calorie labels for food and drink served outside the home improve public health?. BMJ. 2021;372. 10.1136/bmj.n40 33472836

[47] Robinson E, Marty L, Jones A, White M, Smith R, Adams J. Will calorie labels for food and drink served outside the home improve public health?. BMJ. 2021 Jan 20;372. 10.1136/bmj.n40 33472836

[48] Zick A, Wake Y, Reeves S. Nutrition labelling in restaurants: a UK-based case study. Nutr Food Sci 2010;40(6):557–65.

[49] Kant AK, Graubard BI. Eating out in America, 1987–2000: trends and nutritional correlates. Preventive medicine. 2004;38(2):243-9. 10.1016/j.ypmed.2003.10.004 14715218

[50] Darmon N, Drewnowski A. Contribution of food prices and diet cost to socioeconomic disparities in diet quality and health: a systematic review and analysis. Nutrition reviews. 2015;73(10):643 – 60. 10.1093/nutrit/nuv027 PMC4586446 26307238

[51] Maguire ER, Burgoine T, Monsivais P. Area deprivation and the food environment over time: a repeated cross-sectional study on takeaway outlet density and supermarket presence in Norfolk, UK, 1990–2008. Health Place. 2015;1:33:142–7. 10.1016/j.healthplace.2015.02.012 PMC4415115 25841285

[52] Pettigrew S, Rosenberg M, Ferguson R (2013). Consumers‘(in) ability to estimate the energy content of unhealthy foods. Nutrition & Dietetics.

[53] Wansink B, Chandon P. Meal size, not body size, explains errors in estimating the calorie content of meals. Annals of internal medicine. 2006;145(5):326–32. 10.7326/0003-4819-145-5-200609050-00005 16954358

[54] McKinnon RA, Oladipo T, Ferguson MS, Jones OE, Maroto ME, Wolpert B. Reported knowledge of typical daily calorie requirements: relationship to demographic characteristics in US adults. Journal of the Academy of Nutrition and Dietetics. 2019;119(11):1831-41. 10.1016/j.jand.2019.04.024 31296427

[55] Olstad DL, Vermeer J, McCargar LJ, Prowse RJ, Raine KD. Using traffic light labels to improve food selection in recreation and sport facility eating environments. Appetite. 2015;91:329 – 35. 10.1016/j.appet.2015.04.057 25913684

[56] Payne RA, Abel GA. UK indices of multiple deprivation-a way to make comparisons across constituent countries easier. Health Stat Q. 2012;53(22):2015-6.

[57] Urban LE, Dallal GE, Robinson LM, Ausman LM, Saltzman E, Roberts SB. The accuracy of stated energy contents of reduced-energy, commercially prepared foods. Journal of the American Dietetic Association. 2010;110(1):116 – 23. 10.1016/j.jada.2009.10.003 PMC2838242 20102837

[58] Urban LE, McCrory MA, Dallal GE. Calorie counts posted by restaurants are generally accurate; sit-down restaurants underreport compared with fast food. JCOM. 2011;18(9).

