# Supplementary material for: Kilocalorie labelling in the out-of-home sector: an observational study of business practices and consumer behaviour prior to implementation of the mandatory calorie labelling policy in England, 2022
Source: BMC Public Health. 2023 Jun 6;23:1088. doi: 10.1186/s12889-023-16033-8 (PMC10242589; doi:10.1186/s12889-023-16033-8)
Supplement: Supplementary file 1 — Supplementary Material 1 [file 12889_2023_16033_MOESM1_ESM.docx]

**Supplementary Materials**

**Table of contents**

1. Section 1: List of Standard Industrial Classification codes used for sampling 2
2. Section 2: Sample size calculation 3
3. Section 3: Outlets eligible within the four local authorities 4
4. Section 4: Full list of businesses assessed for kcal labelling practices 5
5. Section 5: Measures used to assess kcal labelling based on government guidance 6
6. Section 6: List of survey questions 7
7. Section 7: Missing participant data and missing outlets reported by reason 8
8. Section 8: Kcal labelling assessment including unique businesses 9
9. Section 9: Demographic predictors of kcals purchased, consumed and kcal estimates 10
10. Section 10: Demographic and outlet predictors for levels of noticing of kcal labelling 11
11. Section 11: Supplementary analyses with LA rather than IMD 12

**Section 1: List of Standard Industrial Classification** **codes used for sampling**

Large businesses with the following Standard Industrial Classification (SIC) codes (and hence in the following IDBR sections) that are subject to the policy and thus included in this study:

Section I (accommodation and food service activities)

Within section G:

- - SIC 47.11- Retail sale in non-specialised stores with food, beverages or tobacco predominating.
  - SIC 47.24- Retail sale of bread, cakes, flour confectionery and sugar confectionery in specialised stores.
  - SIC 47.29 Other retail sales of food in specialised stores.

Within section R:

- - SIC 91.03-Operation of historical sites and buildings and similar visitor attractions
  - SIC 91.04-Botanical and zoological gardens and nature reserve
  - SIC 93.11-Operation of sports facilities
  - SIC 93.12-Activities of sports clubs
  - SIC 93.13-Fitness facilities
  - SIC 93.21-Activities of amusement parks and theme parks

Within section J:

- - SIC 59.14-Motion picture projection activities

**Section 2: Sample size calculation based on a pre-post study**

The results presented were based on pre-policy data collection from a larger study examining kcal labelling practices and consumer behaviour pre- and post-mandatory kcal labelling policy implementation in England. Therefore, the sample size was based on a pre/post-study design.

**Kcal labelling practices**

To assess kcal labelling practices, the sample size was determined by 2018 data showing that 17% of large out-of-home food outlets in the UK provided voluntary kcal labelling (Robinson et al, 2018). Based on a paired-proportions sample size calculation, a minimum of N=96 (24 outlets per area) would give 80% power to detect a doubling of prevalence from 17% to 34% at α=0.05 (assuming a pre-post correlation of 0.1).

**Intercept Surveys**

The sample size for the customer intercept surveys was based on results from a Cochrane review which included 28 studies examining the effect of nutritional labelling on purchasing and consumption (Crockett et al, 2018). The sample size was calculated to detect a 47kcal reduction from a baseline mean of 706kcal (SD 326) purchased per individual (7% reduction) as reported in the Cochrane review. Based on an unpaired-means sample size calculation, assuming an intra-class correlation of kcals purchased within outlets of 0.39 (Vartanian et al, 2015) and 10 customers per outlet, we estimated a required sample size of N=3440 pre-post policy from 344 outlets to detect a 7% reduction per individual with 80% power at α=0.05.

**Section 3: Outlets eligible within the four local authorities**

We identified 902 outlets that were eligible within the four local authorities sampled presented in the table 1.

**Supplementary Table 1**

| **all eligible (n=902) in all 4 LAs by business type** | | | |
| --- | --- | --- | --- |
| **bustype** |  | **Freq.** | **Percent (%)** |
| Attractions |  | 6 | 0.7 |
| Sport and entertainment | | 49 | 5.4 |
| Retail |  | 31 | 3.4 |
| Hotels, motels, inns | | 70 | 7.8 |
| Cafes, snack bars |  | 158 | 17.5 |
| Fast food and takeaway | | 210 | 23.3 |
| Pubs, bars and inns | | 212 | 23.5 |
| Restaurants |  | 166 | 18.4 |
| **Total eligible before study** | | **902** | **100** |

**Note:** Outlets highlighted in red were removed prior to sampling due to permission for interviews not being granted.

**Supplementary Table 2 –** Number of Eligible outlets and number and % of outlets sampled for inclusion

| **Business Type** | **Total Eligible** | | | | | | **Sampled** | **%** |
| --- | --- | --- | --- | --- | --- | --- | --- | --- |
|  | **Dudley** | **Liverpool** | **Milton Keynes** | **Richmond** | **Total** | **%** |  |  |
| **Sport and entertainment** | 6 | 14 | 20 | 9 | 49 | 6% | 10 | 3% |
| **Cafes, snack bars** | 15 | 74 | 33 | 36 | 158 | 20% | 66 | 20% |
| **Fast food and takeaway** | 41 | 98 | 53 | 18 | 210 | 26% | 81 | 25% |
| **Pubs, bars and inns** | 49 | 68 | 41 | 54 | 212 | 27% | 92 | 27% |
| **Restaurants** | 19 | 63 | 52 | 32 | 166 | 21% | 81 | 25% |
| **Total** | 130 | 317 | 199 | 149 | **795** | **100%** | **330** | **100%** |

**Section 4: Full list of businesses assessed for kcal labelling practices**

| ASK | Harris and Hoole | Radisson Blu |
| --- | --- | --- |
| Beefeater Grill | Harvester | Reel Cinemas |
| Bella Italia | Hickory's Smokehouse | Revolution |
| Bespoke Hotels Ltd | Hilton Hotels & Resorts | S A Brain |
| Bills | Holiday Inn | Sainsbury's |
| Brasserie Blanc | Joe & The Juice | Shepherd Neame Pubs |
| Brewdog | John Lewis | Showcase Cinemas |
| Brewers Fayre | Kew Gardens | Sizzling Pub |
| Britannia Adelphi Hotel | KFC | Slug & Lettuce |
| Burger King | La Tasca | Soho Coffee Co. |
| Byron | Las iguana | Starbucks Coffee |
| Caffe Nero | Le Bistrot Pierre | Stone house restaurants |
| Campanile Hotels | Leon | Stonegate Pubs |
| Charles Wells Pub Company | M & S | Subway |
| Chiquito | Marriott | T G I Friday's |
| Cineworld | Marston’s | Tesco |
| Costa | McDonald's | The Ivy Cafe |
| Cote | Mercure | The Talbot Hotel |
| Crystal Leisure Ltd | Millennium & Copthorne Hotels | Tortilla |
| Dudley zoo | Miller & Carter | Travelodge |
| Ember Inns | Mitchells & Butler | Turtle Bay |
| Fayre-square | Morrisons Daily | Vintage Inns |
| Fish n chicken | Nando’s | Wagamama |
| Five Guys | Nicholson's Pubs | Walker Art Gallery |
| Franco Manca | Oak Tree Pubs | Wetherspoon |
| Frankie & Benny's | Odeon Cinemas | white 4brasserie |
| Fuller's | Pitcher and piano | Yee Rah |
| Gourmet Burger Kitchen | Pizza Express | YO SUSHI |
| Greene King | Pizza Hut | Young's |
| Hallmark Hotel Group | Premier Inn | Zizzi |

**Section 5: Measures used to assess kcal labelling practices based on government guidance (DHSC, 2018).**

| Kcal labelling Criteria | Rating |
| --- | --- |
| 1) Is kcal labelling provided at any point of choice? (e.g menus, display board) | Y N |
| 2) Is kcal labelling provided at all points of choice? (Menus, display boards etc.) | Y N |
| 3) Is kcal labelling provided for all eligible food items? | Y N |
| 4) Is kcal labelling provided for all non-alcoholic drink items? | Y N |
| 5) Is kcal labelling provided per portion for shareable items? | Y N |
| 6) Is kcal labelling presented close to the item’s name or price? | Y N |
| 7) Is kcal labelling presented as prominently as name or price? | Y N |
| 8) Is kcal reference info for an adult woman displayed anywhere? | Y N |
| 9) Is kcal reference info displayed clearly and prominently? | Y N |

**Section 6: List of survey questions (response options)**

1. What is your age?
2. What gender do you identify as? (Male/ Female/ other)
3. What is your ethnicity? What is the highest degree or level of school you have completed?
4. Can you provide an estimate of the total number of kcals in the food and drink that you purchased for your own consumption?
5. Did you notice the use of kcal labelling in the food outlet? (Yes/No)
6. Did you use the provided kcal labelling when purchasing food and drink? (Yes/No)
7. Why did you use kcal labelling when making your purchases? (To select lower kcal options/ To select higher kcal options/ Other)
8. How did you use kcal labelling to select lower/higher kcal options? (Selected a smaller or larger portion size/ Made a customisation or meal substitution/ Altered choice of food or drink/ Other)
9. What food and drink items did you purchase for your own consumption?
10. Did you make any meal substitutions or customisations? e.g. salad instead of chips? (Yes/No)
11. What customisations/substitutions were made?
12. Did you share any of the food or drink items? (Yes/No)
13. What items were shared?
14. Were any food or drink items leftover and not fully consumed?  (Yes/No)
15. What food or drink items were leftover and not fully consumed?
16. What percentage of the item was leftover and not consumed? Please provide an estimate for each individual food or drink item that was leftover.

**Section 7: Missing participant data and missing outlets reported by reason**

Table 1: Number of participants removed from purchasing, consumption and accuracy of kcal content analyses due to missing data split by outlet type.

|  | Total Participants  (N=856) | Cafes  (N= 39) | Pubs  (N= 377) | Fast-food (N= 27) | Restaurants  (N= 378) | Entertainment venues  (N= 35) |
| --- | --- | --- | --- | --- | --- | --- |
| Nutritional data not provided by the outlet | 802 | 27 | 376 | 9 | 355 | 35 |
| Food items not identifiable | 54 | 12 | 1 | 18 | 23 | 0 |

In total, 856 participants (25.8%) had missing data for analyses examining kcal purchased, consumed and knowledge of kcal content.

**Section 8: Kcal labelling assessment including only unique business**

*Table 2. Frequencies of kcal labelling implemented across all outlets and unique businesses*

| Type | Total outlets (117)  N (%) | Unique Businesses^1^ (90)  N (%) |
| --- | --- | --- |
| Is kcal labelling provided at any point of choice? | 24 (21%) | 18 (20%) |
| Is kcal labelling provided at all points of choice? | 16 (14%) | 11 (12%) |
| Is kcal labelling provided for all food items? | 17 (15%) | 12 (13%) |
| Is kcal labelling provided per portion for sharing menu items? | 10 (9%) | 7 (8%) |
| Is kcal labelling presented close to the item's name and price? | 21 (18%) | 15 (17%) |
| Is kcal labelling presented as prominently as name or price? | 0 (0%) | 0 (0%) |
| Is kcal reference information displayed anywhere? | 11 (9%) | 9 (10%) |
| Is kcal reference information displayed clearly and prominently? | 4 (3%) | 3 (3%) |
| Is kcal labelling provided for all non-alcoholic drink items? | 11 (9%) | 8 (9%) |

*^1^Note: Total outlets = 117 individual outlets from across 90 unique businesses. Unique Businesses = 90 unique businesses each represented by a single outlet*

**Section 9: Demographic predictors of kcal purchased, consumed and kcal estimates**

For the demographic only model, age, gender, ethnicity, and SEP were exposure variables and the number of kcals purchased, consumption (adjusting for leftover estimates and shared items), and customer kcal estimates were outcome variables. The accuracy of kcal estimates, models examining kcal estimates included total kcals purchased to control for meal kcal content. The day of the week the outlet was visited (weekday or weekend) and time of day (midday 12-4 pm or evening 5-9 pm) were additional covariates in these models to control for potential variations in customer eating behaviours based on the time/date of the visit to control for variations in eating habits.

Table 1: Demographic predictors of kcals purchased, consumed and kcal estimates

|  | Kcal Purchased Demographic Model  B [99% CI] | Kcal consumed Demographic Model  B [99% CI] | Kcal estimates Demographic Model  B [99% CI] |
| --- | --- | --- | --- |
| Age | -0.002 [ -2.020; 2.016] | 0.50 [ -1.43; 2.43] | -2.41 [ -3.83 -0.99] * |
| Male (v. Female) | 108 [ 42; 173] * | 140 [ 79 ; 201] * | 69 [21; 118] * |
| None White (v. White) | -155 [-254; -55] * | -129 [-216; -42] * | -42 [-114; 28] |
| Low SEP (v. High) | 24 [ -49; 98] | 43 [ -26; 112] | -74 [-137; -9] * |
| Midday (v. Evening) | -431 [-565; -297] * | -357 [-479; -235] * | -126 [-218; -35] * |
| Weekend (v. Weekday) | 141 [ -23; 304] | 90 [ -57; 237] | 26 [ -65; 117] |
| R^2^ | 0.110 | 0.380 | .449 |
| Adj. R^2^ | 0.108 | 0.376 | .447 |
| Num. obs. | 2446 | 2446 | 2440 |
| N Clusters | 289 | 289 | 289 |

*Table legend: Reference categories (females, white, high SEP, Evening meal, Cafes, IMD1 and labelling absent).* *IMD= Indices of Multiple Deprivation, SEP= Social economic position. Negative values represent an overestimation and positive values represent an underestimation of kcal content.*

**Section 10: Demographic and outlet predictors for levels of noticing of kcal labelling.**

Table 1: Levels of noticing of kcal labelling.

|  | Demographic Model  Log(OR) [95% CI] | Demographic and Outlet Model  Log(OR) [95% CI] |
| --- | --- | --- |
| Age | -0.009 [ -0.019; 0.002] | -0.010 [ -0.022; 0.001] |
| Male (v. Female) | -0.258 [ -0.567; 0.050] | -0.320 [ -0.644; 0.015] |
| None White (v. White) | 0.198 [-0.273; 0.669] | 0.338 [-0.131; 0.808] |
| Low SEP (v. High) | -0.201 [-0.573; -0.171] | -0.308 [-0.681; 0.063] |
| Midday (v. Evening) | -0.173 [-0.791; -0.444] | 0.225 [-0.406; 0.857] |
| Weekend (v. Weekday) | -0.137 [ -0.715; 0.439] | -0.199 [ -0.816; 0.419] |
| Fast Food (v Café) |  | 0.442 [ -0.148; 1.032] |
| Pub (v. Café) |  | 1.330 [0.559; 2.061]* |
| IMD2 (v.IMD1) |  | -0.315 [ -1.133; 0.502] |
| IMD3 (v.IMD1) |  | 0.259 [-0.461; 0.979] |
| IMD4 (v.IMD1) |  | -0.115 [-0.878, 0.648] |
| IMD5 (v.IMD1) |  | 0.008 [-0.664; 0.681] |

*Table legend: Reference categories (females, white, high SEP, Evening Meal, Weekday, Cafes, IMD1 and labelling absent). IMD= Indices of Multiple Deprivation, SEP= Social economic position*

**Section 11: Supplementary analyses with LA rather than IMD in file**

Table 1: Demographic and outlet predictors of kcals purchased.

|  | Demographic Model  B [99% CI] | Demographic and Outlet Model  B [99% CI] |
| --- | --- | --- |
| Age | -0.002 [ -2.020; 2.016] | -2.30 [ -3.89; -0.71] * |
| Male (v. Female) | 108 [ 42; 173] * | 74 [ 17; 132] * |
| None White (v. White) | -155 [-254; -55] * | -80 [-158; -2] * |
| Low SEP (v. High) | 24 [ -49; 98] | 5 [ -57; 66] |
| Midday (v. Evening) | -431 [-565; -297] * | -109 [-225; 6] |
| Weekend (v. Weekday) | 141 [ -23; 304] | 95 [ -8; 199] |
| Entertainment (v. Café) |  | 240 [ 9; 470] * |
| Fast Food (v Café) |  | 278 [ 183; 373] * |
| Pub (v. Café) |  | 947 [ 788; 1107] * |
| Restaurant (v. Café) |  | 730 [ 580; 881] * |
| Liverpool (v. Dudley) |  | -33 [-155; 89] |
| Milton Keynes (v. Dudley) |  | 33 [ -94; 159] |
| Richmond (v. Dudley) |  | -77 [-182; 29] |
| Labelling Present (v. Absent) |  | 37 [ -91; 166] |
| R^2^ | 0.124 | 0.380 |
| Adj. R^2^ | 0.122 | 0.377 |
| Num. obs. | 2446 | 2446 |
| N Clusters | 289 | 289 |

*Table legend: Reference categories (females, white, high SEP, Evening meal, weekday, Cafes, Dudley and labelling absent). SEP= Social economic position*

Table 2: Demographic and outlet predictors of kcals consumed.

|  | Demographic Model  B [99% CI] | Demographic and Outlet Model  B [99% CI] |
| --- | --- | --- |
| Age | 0.50 [ -1.43; 2.43] | -1.92 [ -3.38; -0.47] * |
| Male (v. Female) | 140 [ 79; 201] * | 112 [ 58; 166] * |
| None White (v. White) | -129 [-216; -42] * | -60 [-132; 12] |
| Low SEP (v. High) | 43 [ -26; 112] | 23 [ -34; 79] |
| Midday (v. Evening) | -357 [-479; -235] * | -74 [-172; 24] |
| Weekend (v. Weekday) | 90 [ -57; 237] | 59 [ -31; 150] |
| Entertainment (v. Café) |  | 13 [-174; 200] |
| Fast Food (v Café) |  | 217 [ 131; 303] * |
| Pub (v. Café) |  | 857[ 717; 997] * |
| Restaurant (v. Café) |  | 624 [ 486; 762] * |
| Liverpool (v. Dudley) |  | -50 [-159; 59] |
| Milton Keynes (v. Dudley) |  | 10 [ -97; 118] |
| Richmond (v. Dudley) |  | -86 [-179; 7] |
| Labelling Present (v. Absent) |  | 16 [ -91; 122] |
| R^2^ | 0.110 | 0.380 |
| Adj. R^2^ | 0.108 | 0.376 |
| Num. obs. | 2446 | 2446 |
| N Clusters | 289 | 289 |

*Table legend: Reference categories (females, white, high SEP, Evening meal, weekday, Cafes, Dudley and labelling absent). SEP= Social economic position*

Table 3: Accuracy of participant kcal estimates

|  | Demographic Model  B [95% CI] | Demographic and Outlet Model  B [95% CI] |
| --- | --- | --- |
| Kcals Purchased | -0.56 [ -0.66; -0.46] * | -0.56 [ -0.67; -0.46] * |
| Age | -2.94 [ -4.44; -1.42] * | -1.38 [ -2.86; 0.10] |
| Male (v. Female) | 22 [ -27; 72] | 10 [ -39; 59] |
| None White (v. White) | -46 [-123; 31] | -59 [-131; 14] |
| Low SEP (v. High) | -95 [-159; -31] * | -94 [-157; -31] * |
| Midday (v. Evening) | -139 [-242; -37] * | -103 [-211; 4] |
| Weekend (v. Weekday) | 55 [ -39; 150] | 43 [ -37; 122] |
| Entertainment (v. Café) |  | 87 [ -64; 237] |
| Fast Food (v Café) |  | 194 [ 114; 273] * |
| Pub (v. Café) |  | -21 [-148; 106] |
| Restaurant (v. Café) |  | 329 [ 207; 452] * |
| Liverpool (v. Dudley) |  | 18 [ -80; 117] |
| Milton Keynes (v. Dudley) |  | 46 [ -55; 147] |
| Richmond (v. Dudley) |  | -28 [-112; 55] |
| Labelling Present (v. Absent) |  | 48 [ -40; 136] |
| R^2^ | 0.315 | 0.357 |
| Adj. R^2^ | 0.313 | 0.353 |
| Num. obs. | 2440 | 2440 |
| N Clusters | 289 | 289 |

*Table legend: Reference categories (females, white, high SEP, Evening meal, Weekday, Cafes, Dudley, labelling absent). SEP= Social economic position*

Table 4: Levels of noticing of kcal labelling.

|  | Demographic Model  Log(OR) [95% CI] | Demographic and Outlet Model  Log(OR) [95% CI] |
| --- | --- | --- |
| Age | -0.009 [ -0.019; 0.002] | -0.010 [ -0.022; 0.001] |
| Male (v. Female) | -0.258 [ -0.567; 0.050] | -0.311 [ -0.639; 0.016] |
| None White (v. White) | 0.198 [-0.273; 0.669] | 0.362 [-0.119; 0.845] |
| Low SEP (v. High) | -0.201 [-0.573; -0.171] | -0.306 [-0.703; -0.091] |
| Midday (v. Evening) | -0.173 [-0.791; -0.444] | 0.213 [-0.405; 0.831] |
| Weekend (v. Weekday) | -0.137 [ -0.715; 0.439] | -0.213 [ -0.813; 0.385] |
| Fast Food (v Café) |  | 0.407 [ -0.189; 1.004] |
| Pub (v. Café) |  | 1.357 [0.640; 2.074]* |
| Restaurant (v. Café) |  | 0.394 [ -0.462; 1.250] |
| Liverpool (v. Dudley) |  | -0.424 [-1.087; 0.237] |
| Milton Keynes (v. Dudley) |  | -0.498 [-1.175, 0.178] |
| Richmond (v. Dudley) |  | -0.179 [-0.884; 0.526] |

*Table legend: Reference categories (females, white, high SEP, Evening meal, Weekday, Cafes, Dudley). SEP= Social economic position*
